# Thromboelastometric examination on the ICU before elective procedures

**DOI:** 10.1186/cc13282

**Published:** 2014-03-17

**Authors:** P Lukas

**Affiliations:** 1 University Hospital Motol, Prague, Czech Republic

## Introduction

In critically ill patients a number of elective procedures with a potential risk of bleeding are performed. In this population, coagulation disorder is frequently observed according to the commonly used laboratory parameters of APTT (activated partial thromboplastin time), INR (international normalized ratio) or platelet count. Thromboelastometric examination (ROTEM) evaluates coagulation of whole blood and thus allows the testing of all components of secondary hemostasis.

## Methods

Identification of patients with pathological values of INR, APTT and platelet count before the planned intervention. Through a questionnaire we found a potential correction of coagulopathy carried out by the physician. Performed ROTEM methodology: ExTEM (external pathway thromboelastometry). Inclusion criteria: normal curves and values, CT (clotting time), MCF (maximum clot firmness). In those patients with normal values according to ExTEM examination, no blood products were administered.

## Results

During March 2013 to November 2013, 40 patients were identified as relevant, 26 men of average age 53 years and 14 women of average age 59 years. Central venous cannulation, 12 cases; surgical revision, nine cases; thoracic drainage, six cases; percutaneous endoscopic gastrostomy, five cases; nephrostomy, two cases; epidural catheter, two cases; tracheostomy, two cases; permanent pacemaker, one case; and bronchoscopy, one case. INR normal values were 0.8 to 1.2. The average measured value of INR in men was 1.50, and in women was 1.35. APTT normal values were 0.8 to 1.2. The average measured value of APTT in men was 1.09, and in women was 1.14. Platelet count was 150 × 10^9^/l to 300 × 10^9^/l level of platelets; in men was 252.57, in women was 226.78. ExTEM CT normal values at 41 to 74 seconds: measured value of CT in men was 62.58 and in women was 62.07. MCF normal values were 50 to 72 mm; the measured value of MCF in men was 69.88, and in women was 73.28. Considered administration of blood derivatives: fresh frozen plasma, 123 transfusion units (TU); platelets, 4 TU. In the examined cases no blood products were administered to avoid the risk of bleeding before the elective procedure. No periprocedural bleeding was observed.

## Conclusion

Examination EXTEM in these patients proved to be effective and efficient in predicting bleeding complications in relation with interventions routinely performed on the ICU. Also, these specific cases proved not indicated and thus ineffective administration of blood products.

